# Influence of Lamella Structure on Mechanical Properties and Corrosion Resistance of AlCoCrFeNi_2.1_ Eutectic High-Entropy Alloy Fabricated by Laser-Based Directed Energy Deposition

**DOI:** 10.3390/nano16201285

**Published:** 2026-10-10

**Authors:** Linlin Qin, Chaofei Yang, Yuxiang Zhang, Jiajia Niu, Xinghua Wang

**Affiliations:** 1 Luoyang Ship Material Research Institute, CSSC, Luoyang 471023, China; qll725@163.com (L.Q.);; 2 National Key Laboratory of Marine Corrosion and Protection, Luoyang Ship Material Research Institute, Luoyang 471023, China; 3 School of Materials Science and Engineering, Dalian University of Technology, Dalian 116024, China

**Keywords:** AlCoCrFeNi_2.1_, direct-energy-deposited, lamella thickness, mechanical properties, corrosion resistance

## Abstract

Laser-based directed energy deposition (DED-LB) was employed to fabricate AlCoCrFeNi_2.1_ eutectic high-entropy alloy at laser powers of 1400 W, 1600 W, and 1800 W. All fabricated specimens exhibited a homogeneous alternating FCC and BCC dual-phase microstructure. The FCC lamellae thickness increased from 240.6 to 566.4 nm with increasing laser power from 1400 W to 1800 W, while the BCC increased from 158.6 to 285.0 nm. The hardness, strength, and ductility decreased with increasing laser power. The 1400 W specimen exhibited the optimal combination of mechanical properties, with a hardness of 337 HV, a yield strength of 737 MPa, an ultimate tensile strength of 1185 MPa, and an elongation of 15.0%. Electrochemical measurements in 3.5 wt.% NaCl solution further demonstrated that the 1400 W specimen possessed the best corrosion resistance, exhibiting the lowest corrosion current density of 3.60 × 10^−8^ A cm^−2^ and the most positive corrosion potential of −244.51 mV. Indeed, the strength mechanisms and corrosion resistance are extensively discussed in terms of the lamellar structure during the deposition processes.

## 1. Introduction

High-entropy alloys (HEAs), also referred to as multi-principal-element or compositionally complex alloys, demonstrate excellent comprehensive properties, including superior mechanical properties [1,2], wear resistance [3,4], radiation resistance [5,6], and corrosion resistance [7,8]. Such performance benefits are attributed to the coupled modulation of the high-entropy effect, sluggish diffusion effect, lattice distortion effect, and cocktail effect in the HEAs [9,10,11,12,13,14]. However, single-phase face-centered cubic (FCC) structured HEAs typically exhibit high plasticity and toughness but have relatively low strength, whereas body-centered cubic (BCC) structured HEAs often exhibit high strength and hardness but limited toughness, which severely limits their industrial applications. Lu et al. [15,16] proposed the concept and design method of eutectic high-entropy alloys (EHEAs) to combine the high strength of BCC HEAs and high ductility of FCC HEAs, which comprised a lamella structure with alternating soft FCC and hard BCC phase. Gao et al. [17] reported that the cast AlCoCrFeNi_2.1_ EHEA has an ultimate tensile strength exceeding 1 GPa and a tensile elongation as high as 15.4%, achieving an excellent combination of strength and ductility, which is closely related to its self-formed composite structure at the micrometer scale and the synergistic strengthening effect of nanoscale precipitates within the eutectic phases.

Traditional manufacturing processes face challenges such as severe tool wear, low machining efficiency, and high manufacturing costs when processing high-strength and high-hardness EHEAs materials, which limits their application and promotion. Additive Manufacturing (AM) technology, due to its advantages of being mold-free, near-net shaping, and offering high design freedom, provides a new pathway for the forming and functionalization of EHEAs. Since 2015, Brif et al. [18] successfully prepared the CoCrFeNi HEA using selective laser melting technology and achieved excellent yield strength of 600 MPa, which is much higher than that achieved via traditional manufacturing process (188 MPa). AM has rapidly become a research hotspot due to its potential in exploring the properties of HEAs and their engineering applications. As a widely used AM technique, DED-LB offers several notable advantages, including a high deposition rate, minimal feedstock powder wastage, and excellent near-net-shape forming capability. In particular, DED-LB enables the direct fabrication of target components without geometric constraints. Therefore, combining EHEAs and DED-LB can be a promising method to manufacture intricate parts with high-performance, further expanding the applications of EHEAs in engineering field. Nowadays, many studies on the EHEAs fabricated by DED-LB have been reported [19,20,21]. Compared to traditional casting technology, DED-LB features high temperature gradients and rapid cooling rates, which can induce the formation of finer and more ordered FCC and BCC dual-phase microstructures in eutectic high-entropy alloys. A finer microstructure can lead to a significant increase in strength without a decrease in ductility of the AlCoCrFeNi_2.1_ EHEA alloy [22,23]. In addition, Chen et al. [24] studied the effect of lamellar structures on the fatigue crack initiation and propagation behavior of the AlCoCrFeNi_2.1_ EHEA. The study found that the BCC phase within the FCC phase can effectively delay crack initiation, while the semi-coherent FCC/BCC phase interface acts as a barrier during crack propagation. As the thickness of the BCC lamella decreases, the crack propagation rate significantly reduces, leading to an improvement of fracture toughness in the AlCoCrFeNi_2.1_. For the DED-LB process, the parameters such as the laser power, scanning speed, and scanning strategy can significantly influence the mechanism of microstructure formation, and, consequently, the resulting macroscopic properties. In the research conducted by He et al. [19], it was verified that the interlayer-pause processing strategy can achieve remarkable refinement of the FCC/BCC eutectic lamellar microstructure in AlCoCrFeNi_2.1_ alloy, demonstrating that the microstructure refinement degree of the alloy treated by this optimized process is approximately 40% higher than that of the non-interlayer-pause counterpart. Benefiting from the significantly optimized lamellar microstructure, the modified AlCoCrFeNi_2.1_ alloy delivers an excellent comprehensive mechanical performance, with an ultra-high tensile strength of 1214 MPa and a desirable uniform elongation of 16.3%, outperforming the non-interlayer-pause counterpart by 14% in strength and 47% in uniform elongation.

In the present work, we aim to investigate the influence of laser power on the evolutionary characteristics of FCC/BCC lamellar microstructures of AlCoCrFeNi_2.1_ during the DED-LB process. On this basis, the microstructure–performance correlation between the unique lamellar structural characteristics and mechanical properties is quantitatively established. Furthermore, the work comprehensively clarifies the fracture behavior under uniaxial loading, elucidates the underlying strengthening and toughening mechanisms dominated by FCC and BCC dual-phase lamellar structures during the deposition processes, and explores the electrochemical reaction characteristics of the alloy in corrosive environments.

## 2. Experiment

### 2.1. Specimens Preparation

**Figure 1:**
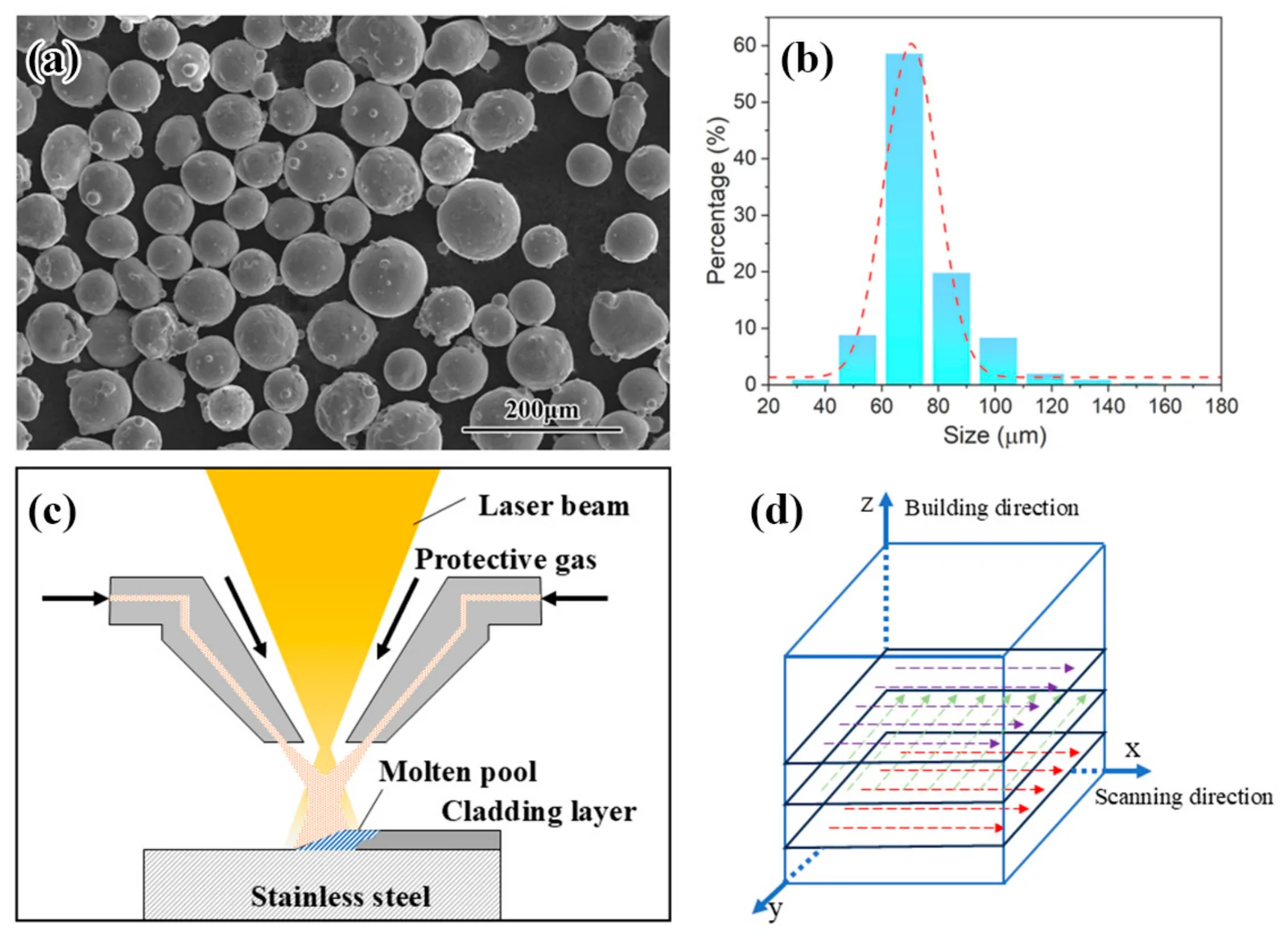
(**a**) A SEM image and (**b**) particle size distribution of AlCoCrFeNi_2.1_ powders, (**c**) schematic diagram of laser cladding process and (**d**) the selected scanning strategy with 90° inter-layer rotation.

Commercially AlCoCrFeNi_2.1_ EHAs powders produced by gas atomization were used as the material for the DED-LB process. The as-received powders exhibited a predominantly near-spherical particles, although satellite particles are also present (Figure 1a), with particle sizes ranging from 53 to 150 μm. The particle size distribution was unimodal with a mean particle size of 69.5 μm (Figure 1b). The chemical composition of the powder is listed in Table 1. Prior to deposition, the as-received powders were dried at 100 °C for 2 h under vacuum to ensure good flowability.

A self-developed four-beam coaxial powder-feeding nozzle coupled with an RC-PGF-D powder feeding system were used. The system comprised a continuous-wave YLS-10000 fiber laser (wavelength λ = 1070 nm, spot diameter 3 mm), an RC-PGF-D coaxial powder-feeding system, a six-axis KUKA robot with its control system, and a work platform. A 316 stainless steel plate was used as the substrate. A scanning strategy with 90° inter-layer rotation between successive layers was adopted for all specimens (as shown in Figure 1d), and the *Z*-axis was defined as the building direction. All depositions were carried out under a high-purity argon (99.99%) atmosphere with a shielding gas flow rate of 15 L/min to minimize oxidation during processing. The final optimized processing parameters are as follows: laser powers of 1400 W, 1600 W, and 1800 W, scanning speed at 14 mm/s, and powder feed rate of 6 g/min. During fabrication, the laser was operated at a defocus distance of 0 mm, and the stand-off distance between the nozzle and the workpiece surface was maintained at 14 mm. Accordingly, cubic specimens measuring 13 mm × 13 mm × 90 mm were built for microstructural characterization and mechanical and corrosion property testing.

### 2.2. Microstructure Characterization

X-ray diffractometer (XRD, Bruker AXS SE, Karlsruhe, Germany) was analyzed using a BRUKER D8 ADVANCE X-ray diffractometer with a Cu radiation target (operated at a voltage of 40 kV and a current of 40 mA) and a LynxEye solid-state detector. XRD measurements were conducted at the central region of each specimen over a 2θ range of 20–100° at a scanning speed of 0.8° min^−1^. The analysis of the BCC and FCC phases were performed using the XRD integrated peak-area method, with the relative phase fraction determined from the integrated diffraction peak areas. The metallographic structures of each specimen were examined with an OLYMPUS GX71 optical microscope (OM). Microstructural features and tensile fracture surface morphologies were characterized using a Quanta 650 scanning electron microscope (SEM, Thermo Fisher Scientific, Hillsboro, OR, USA). Electron backscattered diffraction (EBSD) was employed to analyze phase distribution and grain orientation. Kernel average misorientation (KAM) maps were calculated up to the third nearest neighbor with a maximum misorientation angle of 5° using AZtecCrystal 2.1 software. Prior to microstructural analysis, all the specimens were ground and etched with a solution of 30 vol% aqua regia (HCl: HNO_3_ = 3:1) and 70 vol% deionized water. The specimens for the EBSD were subsequently vibratory polished by 30 nm colloidal silica suspension to remove machining induced subsurface deformation. The specimens for the transmission electron microscope (TEM, JEOL Ltd., Tokyo, Japan) test were ground to a thickness of less than 30 μm and stamped into Φ3 mm discs. The discs were ionthinned using a Gatan PECS II 685 (Gatan, Inc., Pleasanton, CA, USA) and observed by a JEOL 2100P TEM.

### 2.3. Mechanical Property Testing

Microhardness was measured using an HVS-1000A micro-Vickers hardness tester (LaiZhou YuTong Test Instrument Co., Ltd., Yantai, China) with a load of 1.0 kgf and a dwell time of 10 s. The measurements were performed on the XOZ plane parallel to the building direction, with a spacing of 0.4 mm between adjacent indentations, at least 13 indentations were made to ensure statistical reliability. Room-temperature tensile tests were conducted on a 300 kN Material Testing Machine (MTS E45.305-C, MTS Systems (China) Co., Ltd., Shenzhen, China). The tests employed an initial strain rate of 2.5 × 10^−4^/s up to yielding, followed by 6.7 × 10^−3^/s after yielding, respectively. The tensile axis was oriented parallel to the XOZ plane. Round-bar tensile specimens with M10 threaded ends were machined, with a gauge diameter of 5 mm and a gauge length of 25 mm.

### 2.4. Electrochemical Measurements

Electrochemical measurements were performed using a BioLogic SP50e workstation (BioLogic SAS, Seyssinet-Pariset, France) with a conventional three-electrode system in 3.5% NaCl solution at 25 °C. A saturated calomel electrode (SCE) and a platinum electrode were used as the reference and counter electrodes, respectively, while the specimen served as the working electrode. Prior to the electrochemical tests, the open-circuit potential (OCP) test was monitored for 60 min to allow the system to reach a steady state. Electrochemical impedance spectroscopy (EIS) measurements were subsequently conducted over a frequency range from 100 kHz to 0.01 Hz with a perturbation amplitude of 10 mV. Potentiodynamic polarization curves were then recorded at a scan rate of 10 mV/min. All tests were carried out at 25 °C and repeated at least three times under the same conditions to ensure data reproducibility.

## 3. Results

### 3.1. Microstructure Evolution

**Figure 2:**
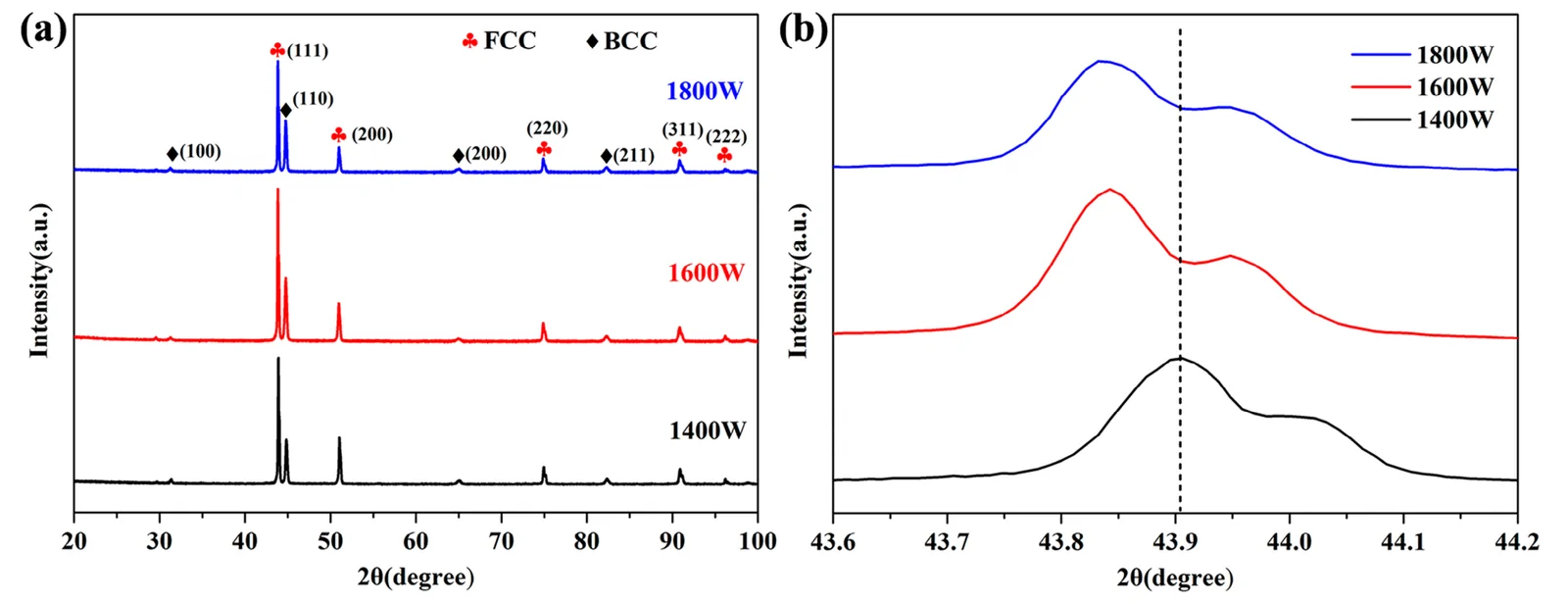
(**a**,**b**) XRD patterns of AlCoCrFeNi_2.1_ EHEA with different laser power.

Figure 2 presents the XRD patterns of the AlCoCrFeNi_2.1_ with different laser powers of 1400 W, 1600 w, and 1800 w, respectively. Figure 2a shows that the diffraction peaks of all samples can be indexed to FCC and BCC phases, indicating that the dual-phase structure is retained over the investigated laser-power range, with no additional crystalline phases detected within the resolution limit of XRD, which is consistent with previous literature [19,22,25,26]. Quantitative phase analysis reveals that the BCC-type phase fraction increases monotonically from 26.6% at 1400 W to 33.2% at 1600 W and further to 42.2% at 1800 W, demonstrating that increasing the laser power promotes the formation or stabilization of the BCC-type phase under the present processing conditions. Higher laser power promotes the diffusion-controlled formation and stabilization of the BCC-type phase by reducing the solidification cooling rate, extending the high-temperature residence of the melt pool and enhancing inter-layer heat accumulation. Figure 2b provides enlarged XRD profiles in the 2θ range of 43.6–44.2°. Compared with the sample fabricated at 1400 W, the low-angle FCC (111) component shifts noticeably toward a lower 2θ value when the laser power is increased to 1600 W, whereas only a limited additional displacement is observed at 1800 W. According to Bragg’s law, an intrinsic shift of the FCC (111) reflection toward a lower diffraction angle corresponds to an increase in the d_(111)_ interplanar spacing and, consequently, an expansion of the FCC lattice parameter. Overall, the XRD results indicate that increasing the laser power simultaneously increases the BCC phase fraction and modifies the lattice state of the FCC phase.

**Figure 3:**
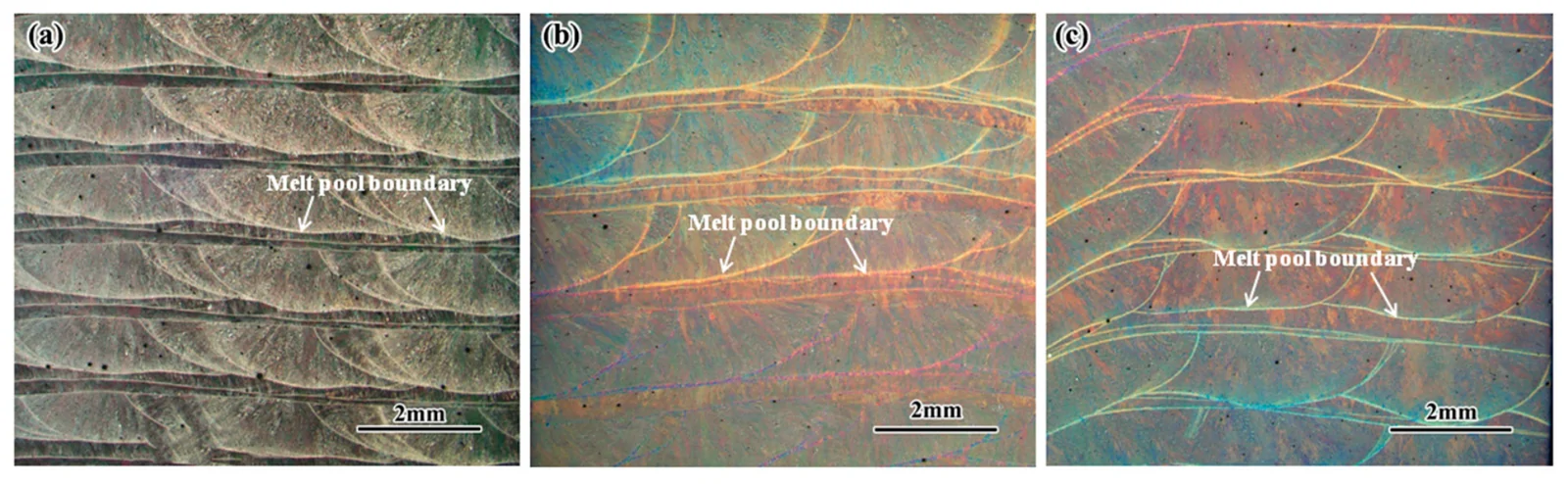
Optical image of XOZ plane with different laser powers of (**a**) 1400 W, (**b**) 1600 W, and (**c**) 1800 W, respectively.

Figure 3 presents the macroscopic morphology of the deposits fabricated with different laser powers of 1400 W, 1600 W, and 1800 W. Under all conditions, a typical fish-scale-like melt pool geometry is observed in the XOZ plane. As the laser power increases, the range of heat conduction and radiation expands, leading to a higher energy absorption rate by the metal powder per unit time and, consequently, more vigorous atomic motion within the melt pool. Once the powder absorbs sufficient energy to form a melt, the surplus energy is dissipated outwards via heat conduction. Higher power gives rise to greater energy dissipation and a wider heat transfer zone, thereby resulting in larger melt pool width and depth. The height of single track is about 0.97 mm, 1.13 mm, and 1.24 mm of the 1400 W, 1600 W, and 1800 W specimens, respectively. Moreover, the AlCoCrFeNi_2.1_ EHEA specimens produced by DED-LB contain a small number of gas pores but are free of cracks. The porosities are approximately 0.33%, 0.38%, and 0.28% for the 1400 W, 1600 W, and 1800 W specimens, respectively. This can be attributed to insufficient time for gas to escape during rapid melt solidification of the molten pool induced by high energy density.

**Figure 4:**
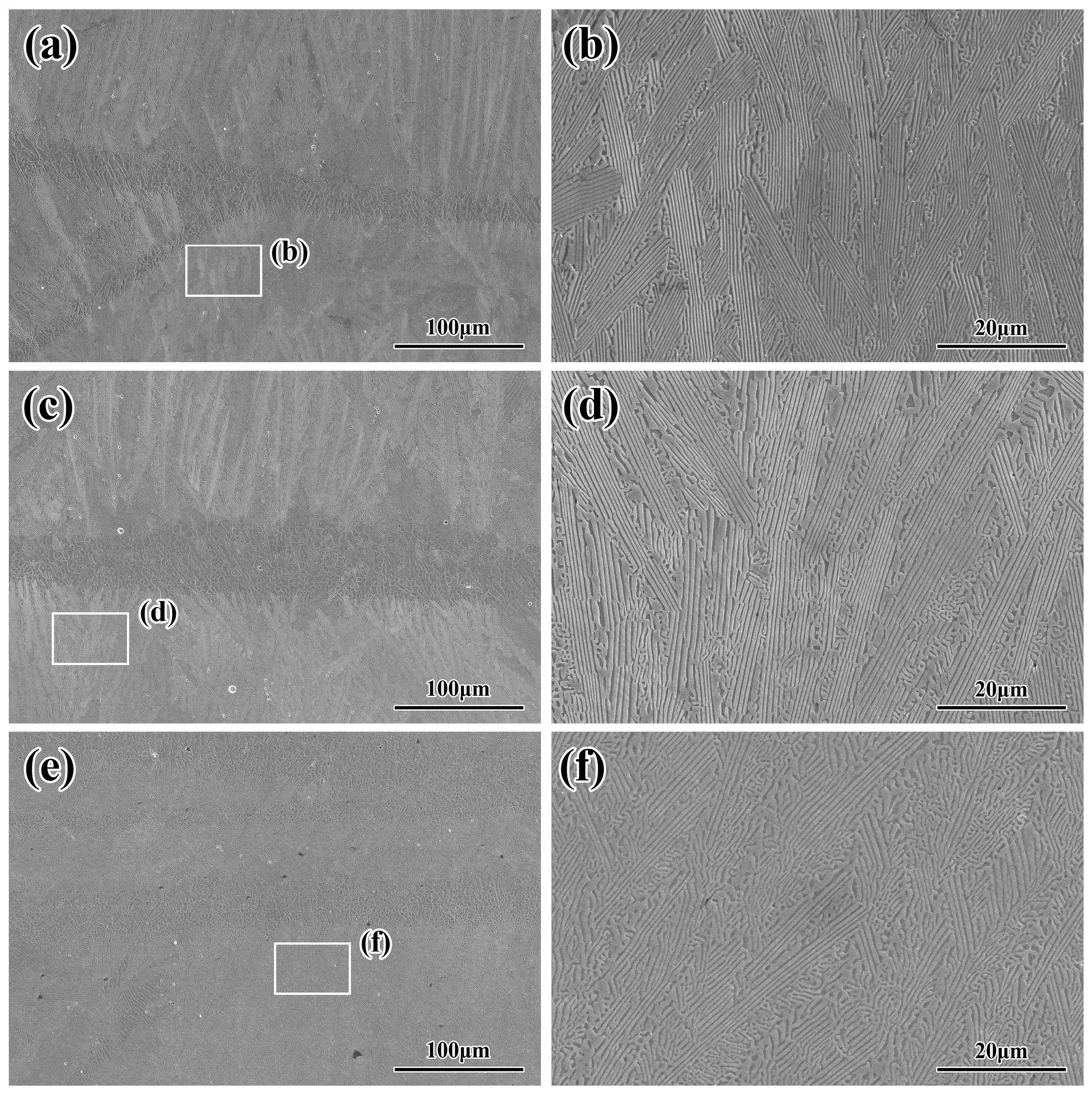
SEM images of the DED-LB AlCoCrFeNi_2.1_ EHEA along the building direction with different laser powers: (**a**,**b**) 1400 W, (**c**,**d**)1600 W and (**e**,**f**) 1800 W. Figure (**b**), (**d**) and (**f**) are the magnified section of Figure (**a**), (**c**) and (**e**), respectively.

**Figure 5:**
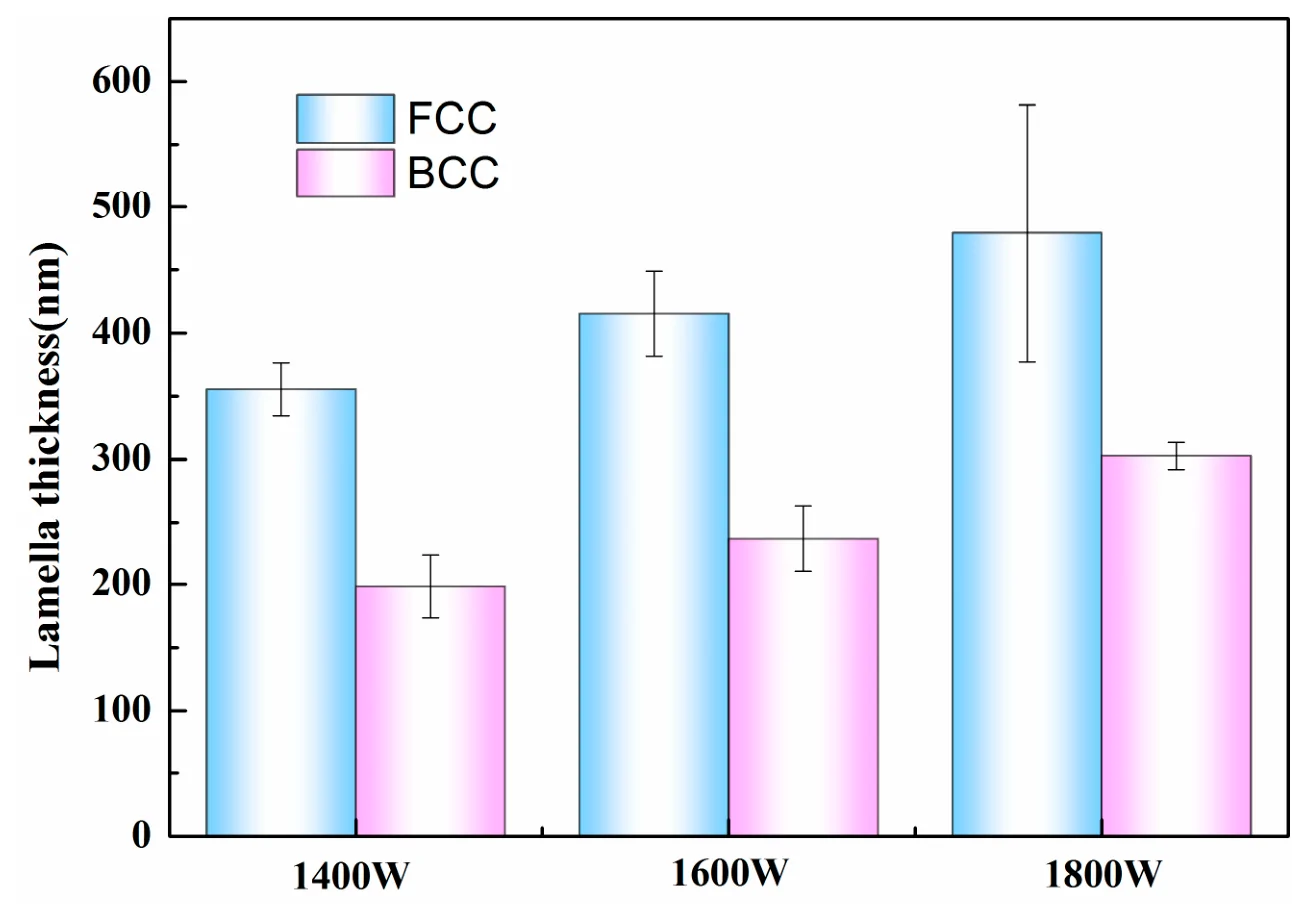
Statistical histograms of the lamella thickness of FCC and BCC phases.

The microstructural morphologies of the specimens fabricated at different laser powers are illustrated in Figure 4. As shown in Figure 4a,c,e, the eutectic structure grew directionally towards the interior of the melt pool, oriented at specific angles or perpendicular to the melt pool boundaries. This orientation is attributed to the tendency of the eutectic growth direction within a melt pool to align with the maximum heat flux direction during the printing process. The higher-magnification images in Figure 4b,d,f reveal a characteristic homogeneous lamellar eutectic microstructure of the DED-LB AlCoCrFeNi_2.1_ alloy, in which the coarse and bright lamellae correspond to the FCC phase and the fine and dark lamellae to the BCC phase. The lamella thicknesses were statistically determined using SEM-based image analysis with the linear intercept method, and the results are summarized in Figure 5. As the laser power increased from 1400 W to 1800 W, the average thickness of the FCC lamellae (λ_FCC_) increased from 240.6 nm to 387.0 nm and 566.4 nm, respectively. Similarly, the average thickness of the BCC lamellae (λ_BCC_) increased from 158.6 nm to 268.2 nm and 285.0 nm. Consequently, the combined lamellar thickness (λ_FCC+BCC_) reached its minimum of 399.1 nm at the lowest laser power of 1400 W.

**Figure 6:**
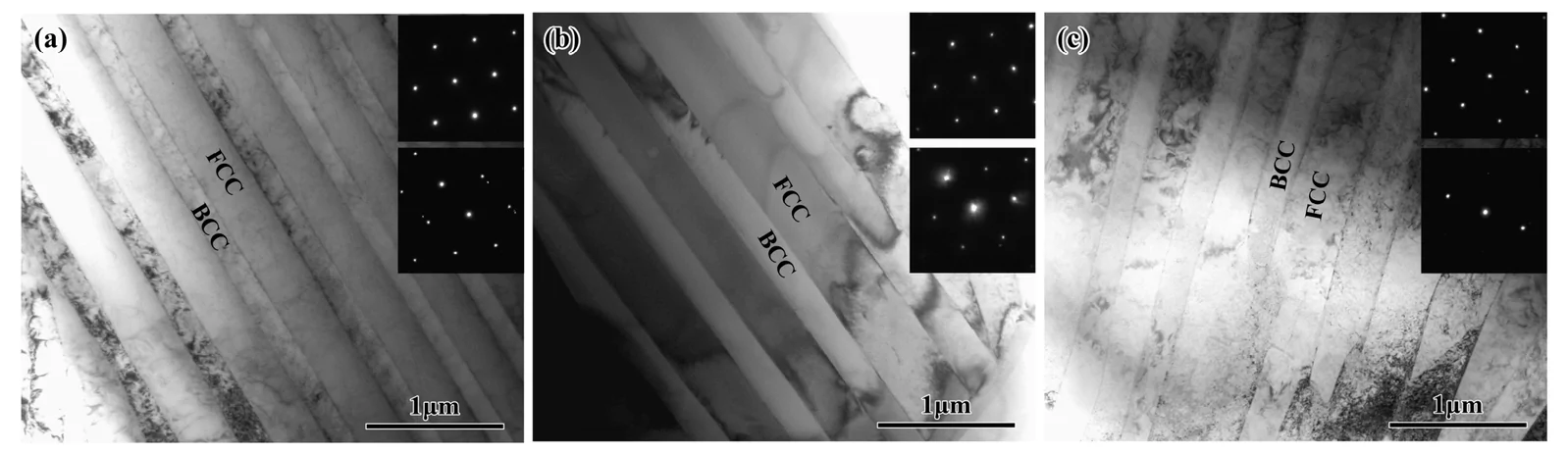
TEM images of the DED-LB AlCoCrFeNi_2.1_ EHEA with different laser powers: (**a**) 1400 W, (**b**)1600 W and (**c**) 1800 W.

Figure 6 shows representative TEM micrographs of the AlCoCrFeNi_2.1_ EHEA fabricated at different laser powers, together with the corresponding selected-area electron diffraction (SAED) patterns. All specimens exhibit a characteristic eutectic lamellar structure and arrange in a well-defined and continuous lamellar configuration. The corresponding SAED patterns further confirm the coexistence of FCC and BCC-type crystal structures. The compositional results summarized in Table 2 reveal pronounced elemental partitioning between the two constituent phases. The FCC phase is preferentially enriched in Co, Cr, and Fe, whereas the BCC phase contains comparatively higher concentrations of Al and Ni. This compositional partitioning is consistent with the thermodynamic tendency for phase separation in AlCoCrFeNi_2.1_ alloy. Consequently, the eutectic microstructure can be described as a chemically partitioned dual-phase lamellar structure consisting of Co-Cr-Fe rich FCC lamellae and Al-Ni rich BCC lamellae.

**Figure 7:**
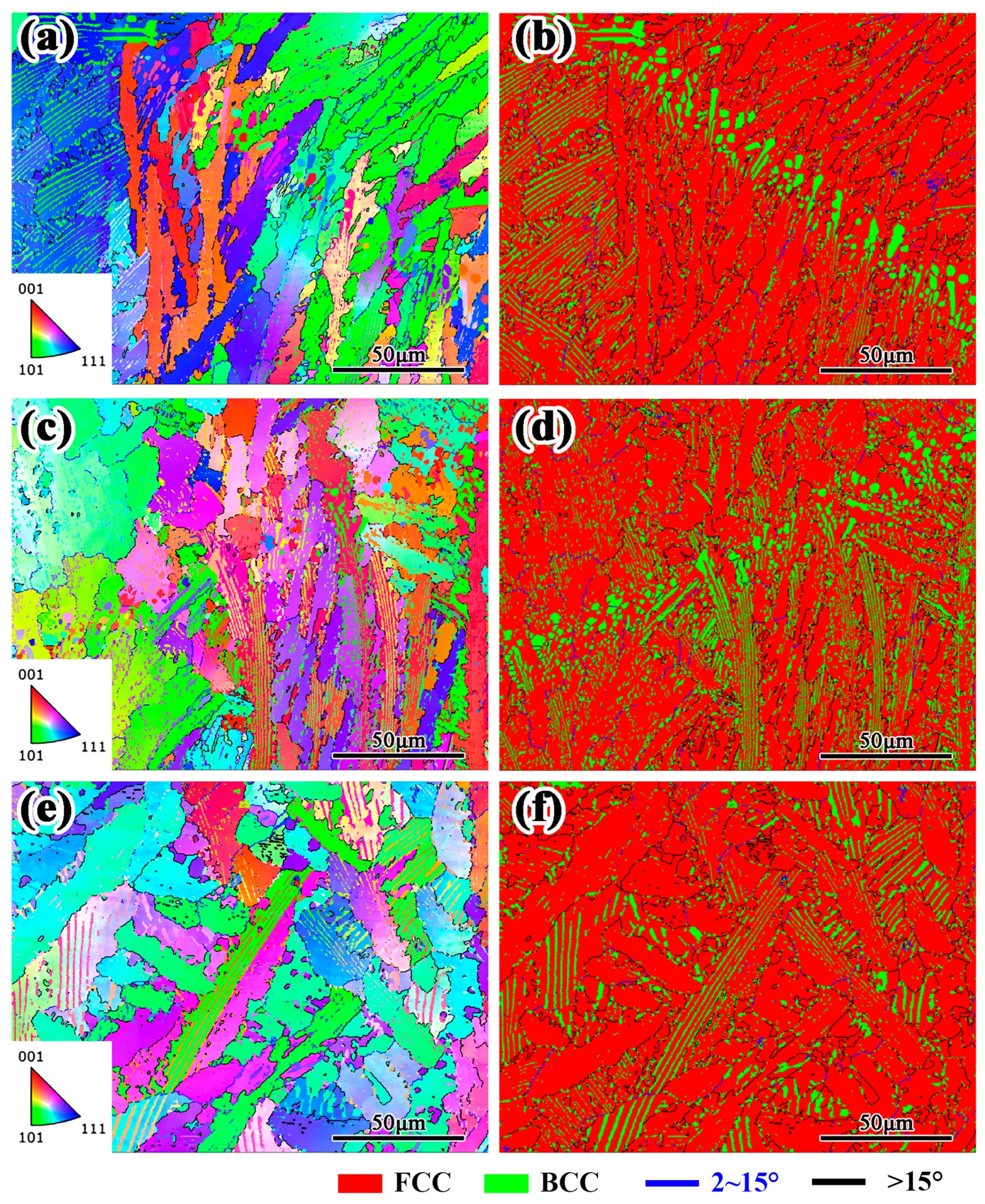
IPF and corresponding phase images of XOZ plane with different laser powers: (**a**,**b**) 1400 W, (**c**,**d**) 1600 W and (**e**,**f**) 1800 W.

Figure 7 presents the microstructures observed in the XOZ plane. The inverse pole figure (IPF) maps reveal that the grains are predominantly large columnar grains that grew along the heat flow direction. A distinct variation in the morphology and characteristic size of the columnar grains can be observed with increasing laser power, as shown in Figure 7a,c,e. The microstructure of the 1400 W specimen is dominated by relatively fine and highly elongated columnar grains with a comparatively high aspect ratio. When the laser power is increased to 1600 W, the columnar grains become progressively wider and coarser, accompanied by a reduction in their aspect ratio. At 1800 W, further grain coarsening is evident, and the grain morphology becomes less elongated and more irregular. The corresponding phase distribution maps for the specimens fabricated at different laser powers are shown in Figure 7b, d and f, respectively. Combined analysis of the phase maps and IPF maps indicates that the matrix consists of FCC grains, within which BCC phase domains are regularly distributed. Uncalibrated regions are also present, exhibiting a non-random orientation. This arises because EBSD tends to misidentify the BCC phase as FCC when the lamellar thickness approaches the resolution limit of the technique.

### 3.2. Mechanical Properties

**Figure 8:**
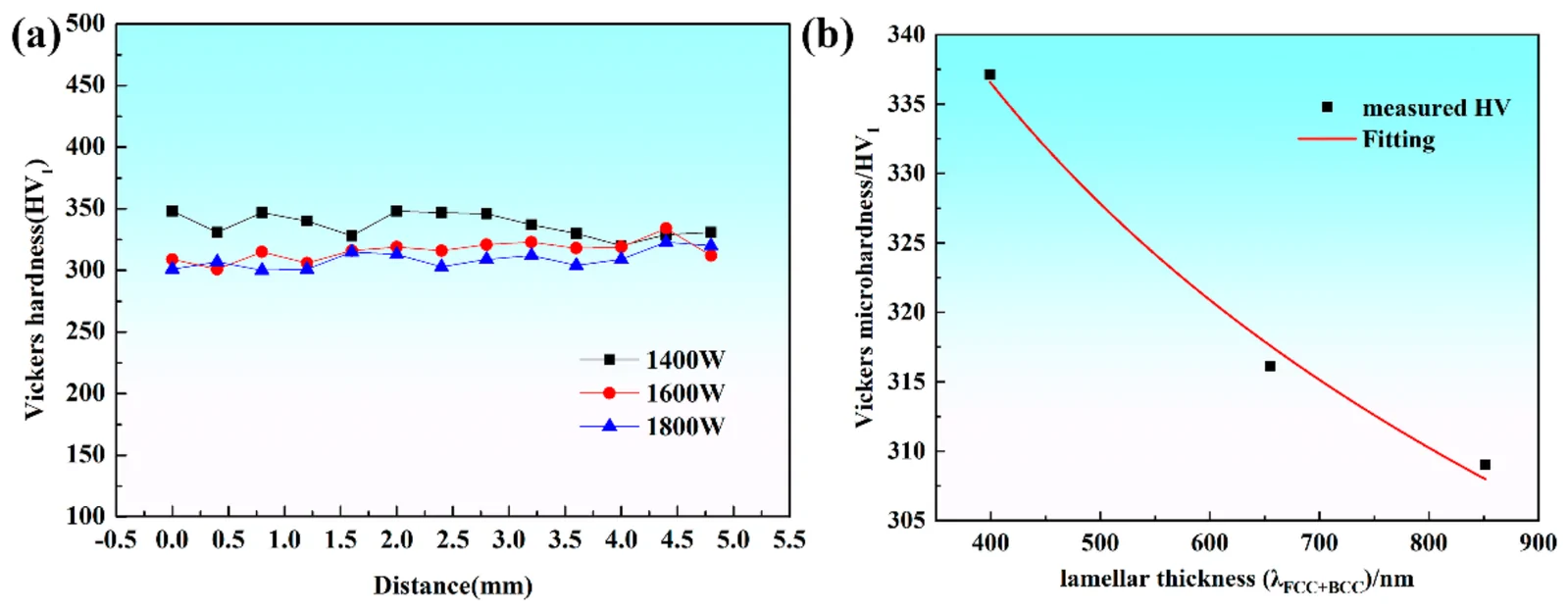
(**a**) The microhardness curves along scanning direction and (**b**) Relationship between microhardness and characteristic lamellar spacing of AlCoCrFeNi_2.1_ EHEA.

Figure 8 shows the Vickers hardness distributions of the AlCoCrFeNi_2.1_ EHEA measured along the building direction under different laser powers. As shown in Figure 8a, the hardness values obtained under each processing condition fluctuate within a relatively narrow range over the investigated distance, without exhibiting an evident monotonic variation or pronounced hardness gradient. This behavior indicates a comparatively uniform mechanical response along the building direction. Nevertheless, a systematic downward shift in the hardness profiles is observed with increasing laser power. Figure 8b further correlates the average microhardness with the characteristic lamellar thickness, λ_FCC+BCC_. The average microhardness decreases monotonically from 337.1 HV at 1400 W to 316.1 HV at 1600 W and further to 309.0 HV at 1800 W, and the experimental data are well described by a Hall–Petch type power-law fitting relationship of HV = 679.5λ^−0.117^ (λ in μm, R^2^ = 0.992), which demonstrates a strong inverse correlation between hardness and lamellar spacing.

**Figure 9:**
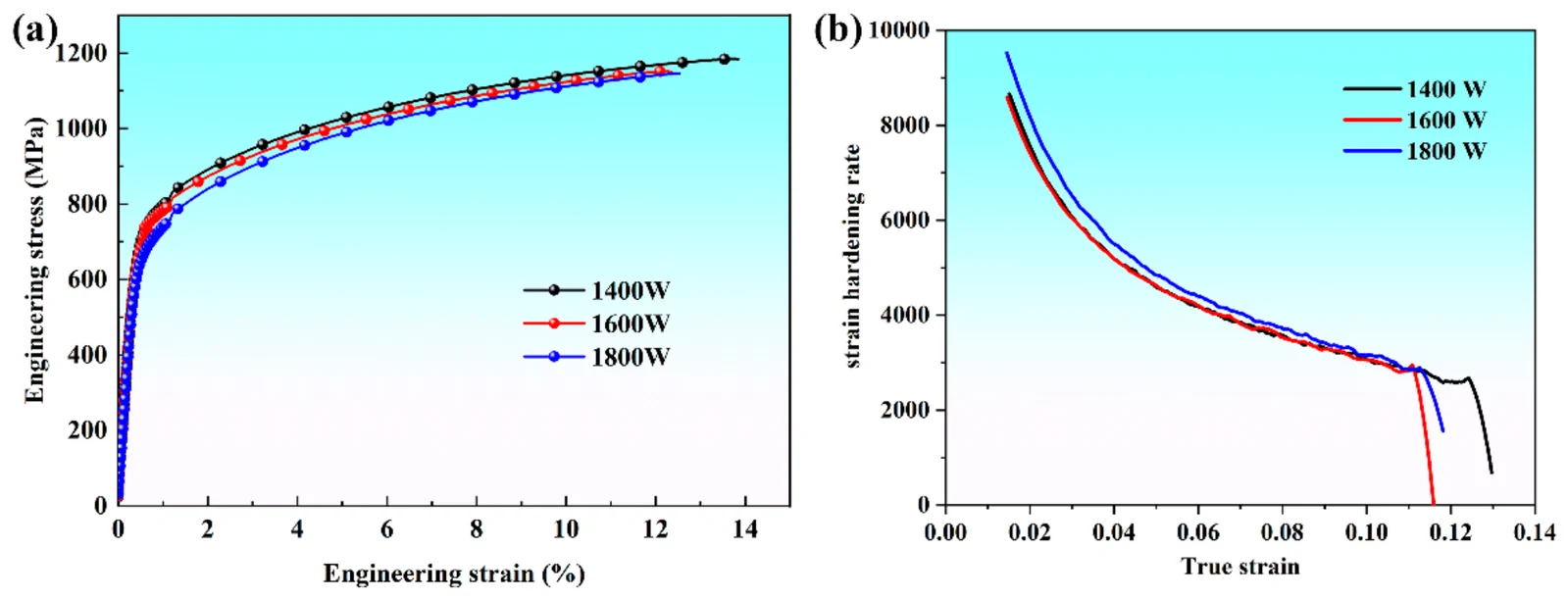
Mechanical response of AlCoCrFeNi_2.1_ EHEA specimens fabricated at different laser powers: (**a**) engineering stress–strain curves and (**b**) strain-hardening-rate curves as a function of true strain.

Figure 9a presents the engineering stress–strain curves of the AlCoCrFeNi_2.1_ EHEA specimens fabricated at different laser powers. As the laser power increased, both the ultimate tensile strength (UTS) and yield strength of the alloy decreased gradually. The specimen fabricated at 1400 W exhibits the optimal combination of strength and ductility, with a yield strength of 737 MPa, an UTS of 1185 MPa, and an elongation to failure of 15.0%. When the laser power is increased to 1600 W, the yield strength, UTS, and elongation decrease to 705 MPa, 1152 MPa, and 13.5%, respectively. A further increase in laser power to 1800 W leads to corresponding values of 703 MPa, 1145 MPa, and 12.0%. Relative to the 1400 W condition, the specimen fabricated at 1800 W shows decreases of approximately 4.6% in yield strength, 3.4% in UTS, and 20.0% in elongation. Figure 9b shows the corresponding strain-hardening-rate curves. For all specimens, the strain-hardening rate decreases continuously with increasing true strain, reflecting the progressive reduction in the net dislocation-storage rate during plastic deformation. Although the 1800 W specimen exhibits a slightly higher strain-hardening rate at the early stage of plastic deformation, the three curves become comparable over most of the uniform deformation regime. More importantly, the 1400 W specimen sustains a positive strain-hardening response to a larger true strain, whereas the hardening capacity of the 1600 and 1800 W specimens terminates earlier. This more persistent strain-hardening capability delays plastic instability and strain localization, consistent with the higher tensile ductility of the 1400 W specimen. Overall, the tensile results are consistent with the microhardness trend, confirming that a lower laser power of 1400 W provides a more favorable thermal history and microstructural state, thereby achieving the best strength–ductility synergy among the investigated processing conditions.

### 3.3. Fracture Morphologies

**Figure 10:**
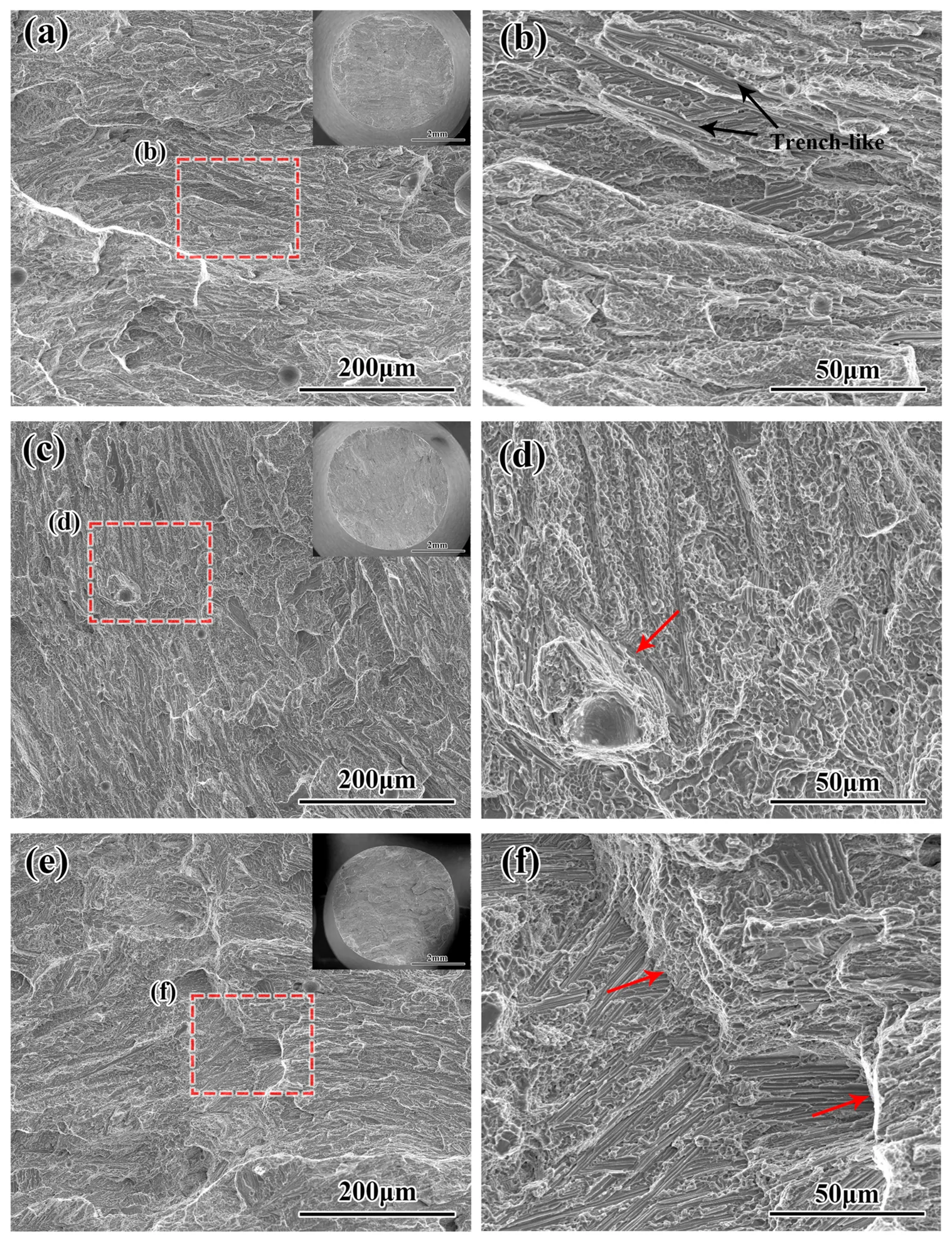
Tensile fracture morphologies of tensile fracture of DED-LB specimens with different laser powers: (**a**,**b**) 1400 W, (**c**,**d**) 1600 W and (**e**,**f**) 1800 W.

Figure 10 presents the tensile fracture morphologies of the specimens fabricated at different laser powers of 1400 W, 1600 W and 1800 W. Increasing the laser power causes the fracture mode to evolve from a ductile-dominated mixed fracture at 1400 W toward a quasi-cleavage and intergranular dominated mixed ductile-brittle fracture at higher laser powers. For the specimen fabricated at 1400 W, the fracture surface is highly tortuous and contains abundant fine dimples, pronounced tearing ridges, and elongated trench-like features (as shown by the black arrows), as shown in Figure 10a,b. At 1600 W, the fracture surface still contains locally dimpled regions and tearing ridges. However, relatively flat quasi-cleavage facets and river-like markings become more apparent, as shown in Figure 10c,d. The reduced fraction and less uniform distribution of ductile dimples indicate a diminished capacity for plastic accommodation before final fracture. Moreover, the increased occurrence of quasi-cleavage features suggests that crack propagation becomes less tortuous and more readily proceeds through the brittle BCC constituent or along the FCC/BCC interfaces. With a further increase in laser power to 1800 W, the fracture surface exhibits more extensive cleavage-like and intergranular regions (as shown by the red arrows), and pronounced trench-like features, whereas the number and depth of ductile dimples are reduced, as illustrated in Figure 10e,f. These observations indicate a further suppression of plastic deformation and an enhanced tendency toward quasi-cleavage and intergranular fracture.

**Figure 11:**
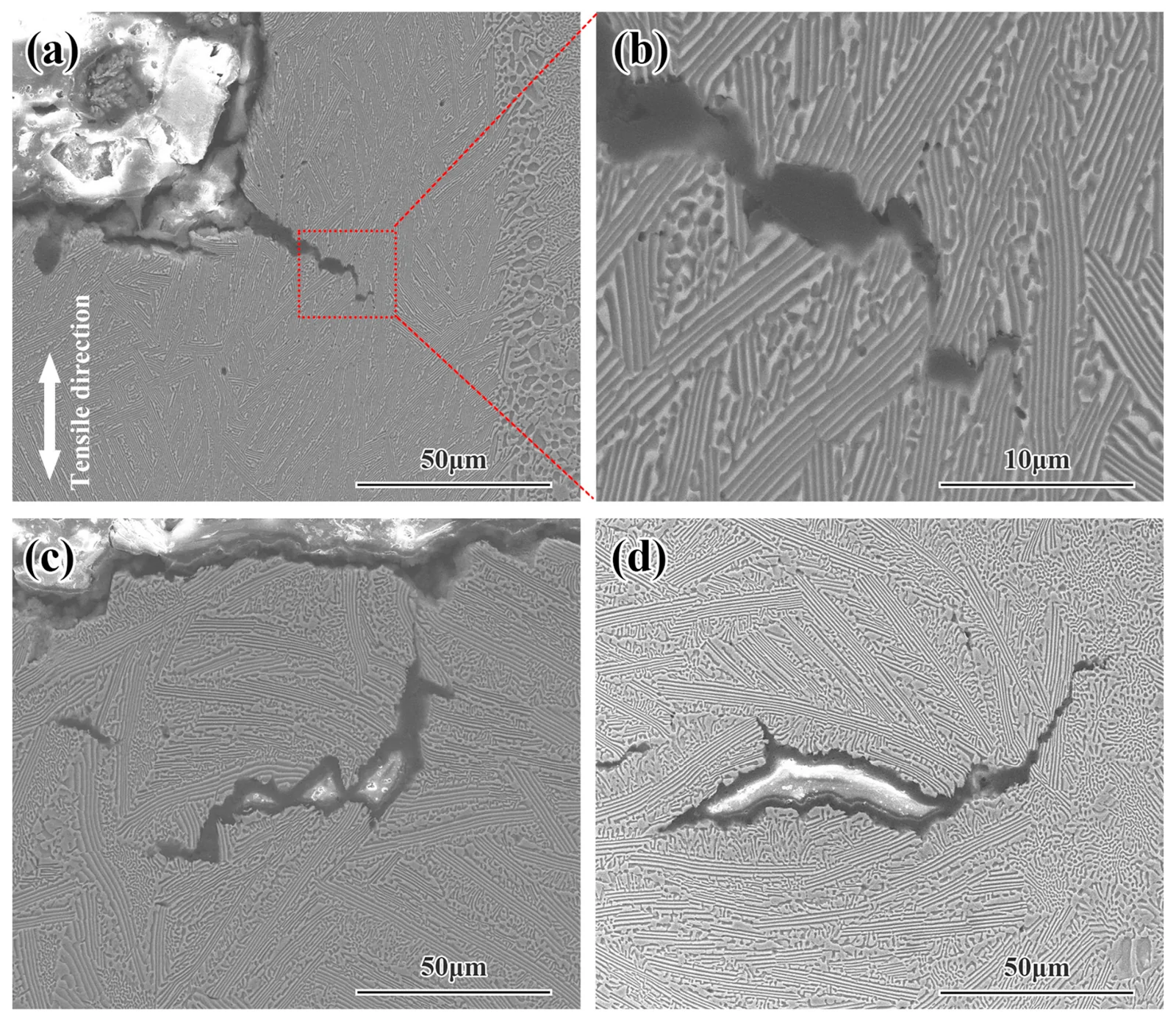
SEM micrographs of longitudinal sections adjacent to the tensile fracture surfaces of the AlCoCrFeNi_2.1_ EHEA fabricated at different laser powers: (**a**) 1400 W, (**c**) 1600 W, and (**d**) 1800 W. Figure (**b**) enlarged view of the region marked by the red dashed rectangle in (**a**).

Figure 11 shows the SEM images of the longitudinal cross-sections adjacent to the tensile fracture surfaces of the AlCoCrFeNi_2.1_ fabricated at laser powers of 1400 W, 1600 W, and 1800 W. For the specimen fabricated at 1400 W, the secondary cracks are relatively short, discontinuous, and highly tortuous, as shown in Figure 11a,b. At higher magnification, the crack path is observed to intersect the eutectic lamellae and propagate predominantly through the interior of the FCC/BCC eutectic lamellae, indicating that transgranular cracking is the dominant failure mode. These characteristics are consistent with the abundant dimples and tearing ridges observed on the corresponding fracture surface and account for the superior tensile ductility of the 1400 W specimen. In contrast, the specimens fabricated at 1600 W and 1800 W exhibit wider and more continuous secondary cracks, as illustrated in Figure 11c,d. The cracks show an increasing tendency to propagate along eutectic-colony and grain boundaries rather than penetrating directly through the colony interiors, indicating a progressive increase in the contribution of intergranular fracture, which correspond to the secondary cracks passing through the sample cross-section on the macroscopic fracture surfaces (Figure 10). In particular, the crack observed in the 1800 W specimen follows a comparatively continuous boundary-dominated path, suggesting reduced resistance to crack propagation.

### 3.4. Electrochemical Corrosion

**Figure 12:**
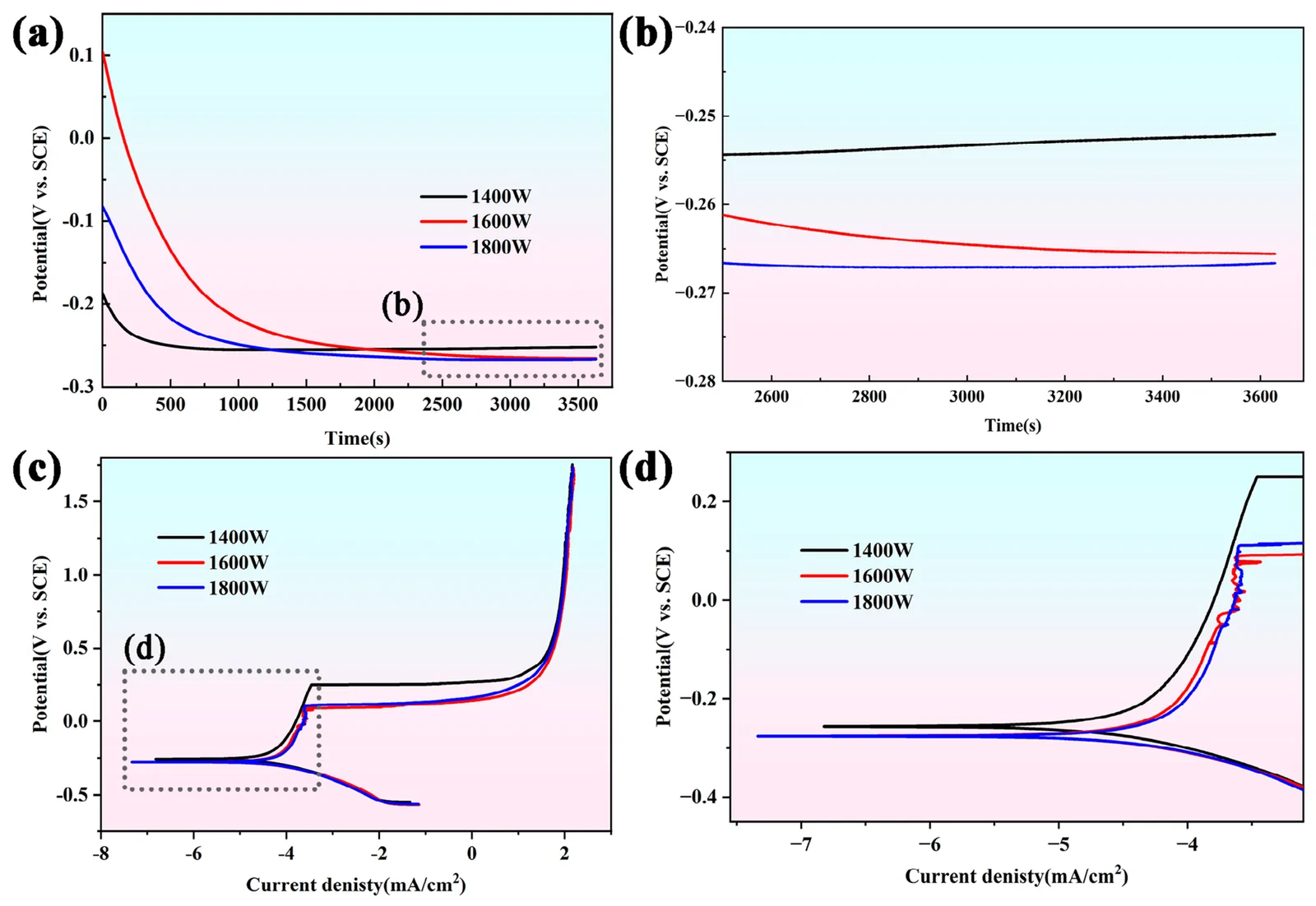
(**a**,**c**) OCP and potentiodynamic polarization curves of AlCoCrFeNi_2.1_ EHEA in 3.5% NaCl solution. Figure (**b**) and (**d**) are the locally enlarged views of Figure (**a**) and (**c**), respectively.

To elucidate the influence of laser power and the resulting FCC/BCC eutectic microstructure on the corrosion behavior of the AlCoCrFeNi_2.1_ EHEA, electrochemical measurements were conducted in a 3.5 wt.% NaCl solution. Figure 12a,b present the OCP of the specimens fabricated at laser powers of 1400, 1600, and 1800 W. For all specimens, the OCP initially shifted rapidly in the negative direction and subsequently approached a quasi-steady state after prolonged immersion. The initial negative displacement may be associated with the dissolution or local breakdown of the native surface oxide upon exposure to the chloride-containing electrolyte, whereas the subsequent stabilization reflects the establishment of a dynamic equilibrium between passive-film formation and localized dissolution. The specimen fabricated at 1400 W exhibited the most positive steady-state OCP, approximately −253 mV. In comparison, the steady-state potentials of the 1600 and 1800 W specimens were approximately −265 mV and 267 mV. The 1400 W specimen shows a slightly more noble OCP.

The potentiodynamic polarization curves and their enlarged views are shown in Figure 12c,d, which reflects the cathodic reduction and anodic dissolution processes, respectively. Upon anodic polarization, the current density initially increases and then reaches a relatively low-current quasi-passive region, indicating surface-film formation. A subsequent sharp increase in anodic current at higher potentials suggests progressive film destabilization and accelerated dissolution. The corresponding electrochemical parameters obtained by Tafel extrapolation are summarized in Table 3. The 1400 W specimen exhibited a corrosion potential (Ecorr) of −244.51 mV and a corrosion current density (Icorr) of 3.60 × 10^−8^ A⋅cm^−2^. The polarization responses of the specimens fabricated at 1600 and 1800 W were relatively similar, with only minor differences in Ecorr and Icorr. Nevertheless, both specimens exhibited more negative corrosion potentials and higher corrosion current densities than the 1400 W specimen, demonstrating comparatively inferior corrosion resistance. The consistency between the steady-state OCP values and the polarization parameters confirms that the specimen fabricated at 1400 W possesses the most favorable electrochemical stability among the three processing conditions. Among the three alloys, the one fabricated at 1400 W demonstrated the most superior corrosion resistance.

**Figure 13:**
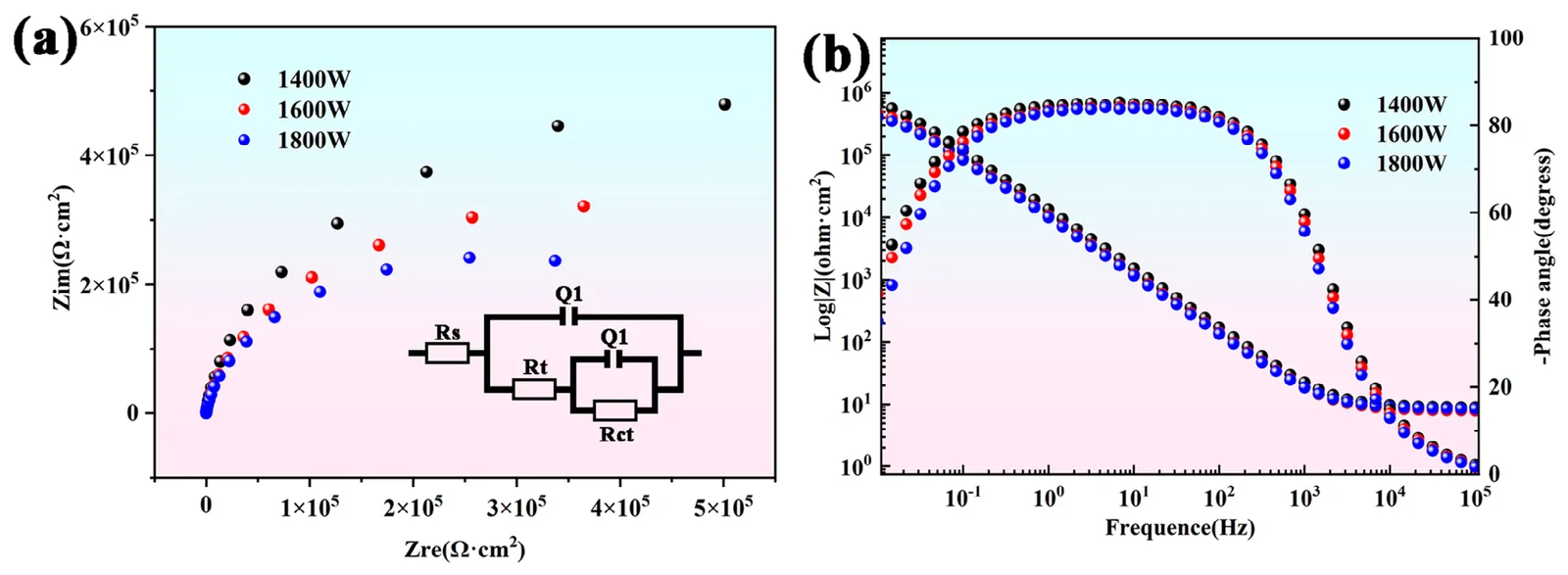
EIS results of the AlCoCrFeNi_2.1_ EHEA fabricated at different laser powers in a 3.5 wt.% NaCl solution: (**a**) Nyquist plots and the equivalent electrical circuit used for data fitting; (**b**) Bode impedance-modulus and phase-angle plots.

EIS was further employed to evaluate the corrosion behavior and passive-film characteristics of the AlCoCrFeNi_2.1_ EHEA fabricated at different laser powers. As shown in Figure 13a, all specimens exhibit depressed and incomplete capacitive arcs in the Nyquist plots. Despite their similar overall spectral features, the specimens display noticeable differences in the dimensions of the capacitive arcs. The arc diameter decreases in the order of 1400 W > 1600 W > 1800 W, suggesting a progressive reduction in the overall interfacial resistance with increasing laser power. Accordingly, the specimen fabricated at 1400 W exhibits the highest impedance and the greatest resistance to electrochemical charge transfer, in agreement with the OCP and potentiodynamic polarization results. Figure 13b presents the corresponding Bode impedance-modulus and phase-angle plots. In the low-frequency region, the impedance modulus, ∣Z∣, follows the same descending order of 1400 W > 1600 W > 1800 W. Since the low-frequency impedance response is closely related to the overall polarization resistance of the electrode, the higher impedance modulus ∣Z∣ value of the 1400 W specimen indicates a lower corrosion rate and a more protective surface film. By comparison, the lower low-frequency impedance moduli of the 1600 W and 1800 W specimens reflect an increased susceptibility to interfacial electrochemical reactions. The phase-angle curves exhibit broad capacitive maxima extending over a wide frequency range, with a weak shoulder or overlapping relaxation response, indicating the presence of at least two electrochemical time constants. The high-frequency response can be associated predominantly with the dielectric behavior of the surface passive film, whereas the medium- and low-frequency response is related to charge-transfer processes occurring at the passive-film/substrate or electrode/electrolyte interface. The broad phase-angle distribution further indicates that these two relaxation processes partially overlap and cannot be completely distinguished within the investigated frequency range. Among the three deposited conditions, the 1400 W specimen exhibits a slightly broader high-phase-angle region and maintains a higher impedance response, implying a more homogeneous and protective passive layer. In contrast, the reduced phase-angle response of the specimens fabricated at higher laser powers suggests increased interfacial heterogeneity and a greater contribution from electrochemical dissolution.

To quantitatively characterize the electrochemical processes, the EIS spectra were fitted using the equivalent electrical circuit shown in the inset of Figure 12a, which may be expressed as R_s_ − (Q_1_ ∥R_f_) − (Q_2_ ∥R_ct_). In this model, R_s_ represents the solution resistance; R_t_ denotes the resistance associated with ionic transport through defects or conductive paths in the passive film; and R_ct_ represents the charge-transfer resistance at the electrochemically active interface. The constant-phase elements Q_1_ and Q_2_ describe the non-ideal capacitive responses of the passive film and the electrical double layer, respectively. The use of constant-phase elements rather than ideal capacitors accounts for the compositional, structural, and topographical heterogeneity of the alloy surface. The fitted parameters are summarized in Table 4. Both R_t_ and R_ct_ decrease with increasing laser power, demonstrating that the specimen fabricated at 1400 W possesses not only a more resistive passive film but also a greater resistance to interfacial charge transfer. Therefore, the higher R_t_ and R_ct_ of the 1400 W specimen confirm its superior corrosion resistance. Conversely, the lower resistance values obtained for the 1600 and 1800 W specimens indicate that their passive films provide less effective protection against chloride-induced electrochemical attack.

## 4. Discussions

### 4.1. The Fracture and Strength Mechanisms

**Figure 14:**
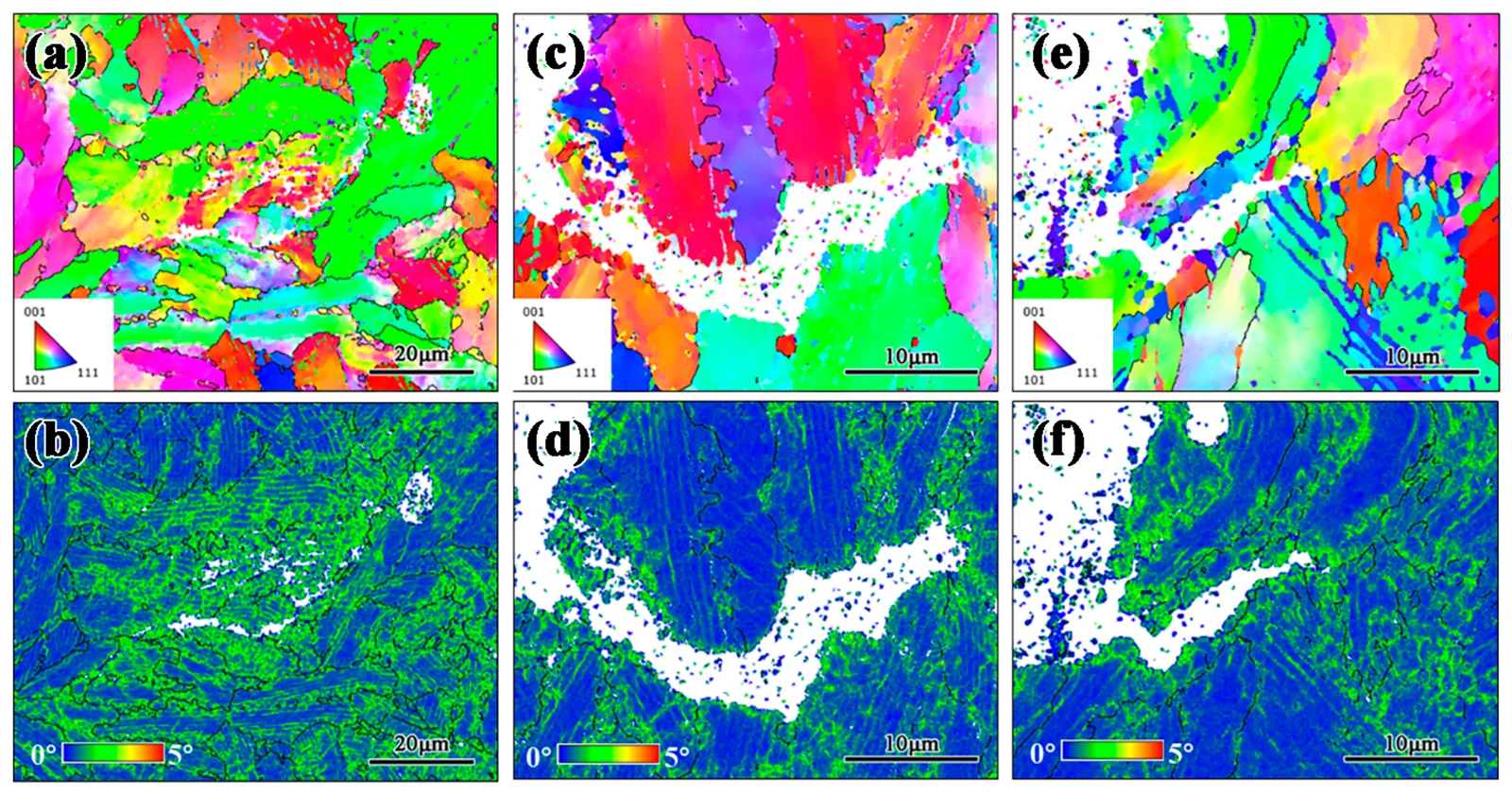
Inverse pole figure orientation maps and corresponding KAM maps of the regions containing secondary cracks in the tensile-deformed AlCoCrFeNi_2.1_ specimens fabricated at different laser powers: (**a**,**b**) 1400 W, (**c**,**d**) 1600 W, and (**e**,**f**) 1800 W.

To further clarify the role of the FCC and BCC lamellar structure in crack initiation and propagation in the AlCoCrFeNi_2.1_ alloy, post-mortem characterization was conducted on the longitudinal side surfaces adjacent to the tensile fracture regions of the AlCoCrFeNi_2.1_ specimens fabricated at different laser powers, as shown in Figure 14. For the specimen fabricated at 1400 W, only relatively short and discontinuous intergranular cracks were observed, as shown in Figure 14a. The crack paths were highly tortuous and frequently deflected by changes in grain orientation. In contrast, the specimens fabricated at 1600 W and 1800 W contained longer and more continuous secondary cracks that preferentially propagated along high-angle grain boundaries, as shown in Figure 14c,e. This observation indicates that increasing the laser power promotes a transition from predominantly transgranular or highly deflected crack propagation toward boundary-assisted cracking. Such a transition is consistent with the fracture-surface and longitudinal-section observations in Figure 10 and Figure 11, where the fraction of quasi-cleavage and intergranular fracture features increased progressively with laser power. The corresponding KAM maps in Figure 14b,d,f reveal pronounced spatial heterogeneity in the local lattice rotation around the secondary cracks. In the 1600 and 1800 W specimens, crack initiation and propagation occurred preferentially in regions exhibiting strain concentrated, particularly near high-angle grain boundaries. These localized high-KAM regions indicate the accumulation of geometrically necessary dislocations and severe strain incompatibility between neighboring grains or constituent phases. Once the local plastic-strain accommodation capacity was exceeded, these regions became preferential sites for microcrack nucleation. The subsequent linkage of interfacial and intergranular microcracks resulted in the formation of the long secondary cracks observed at higher laser powers.

A different deformation response was observed in the 1400 W specimen. Although its overall KAM level was higher, the lattice curvature was distributed more extensively throughout the refined eutectic structure rather than being concentrated exclusively at a limited number of grain boundaries. The high density of finely spaced FCC/BCC interfaces provided numerous sites for dislocation storage and strain accommodation, thereby distributing the plastic incompatibility over a larger interfacial area [23,26,27,28,29]. Consequently, severe strain localization at individual boundaries was alleviated, which delayed the nucleation and coalescence of long secondary cracks. Therefore, a high spatially averaged KAM value does not necessarily indicate more severe local damage, rather, the spatial distribution of KAM is critical. A broadly distributed misorientation field can reflect effective dislocation storage and homogeneous plastic accommodation, whereas sharply localized KAM maxima are more closely associated with crack initiation.

**Figure 15:**
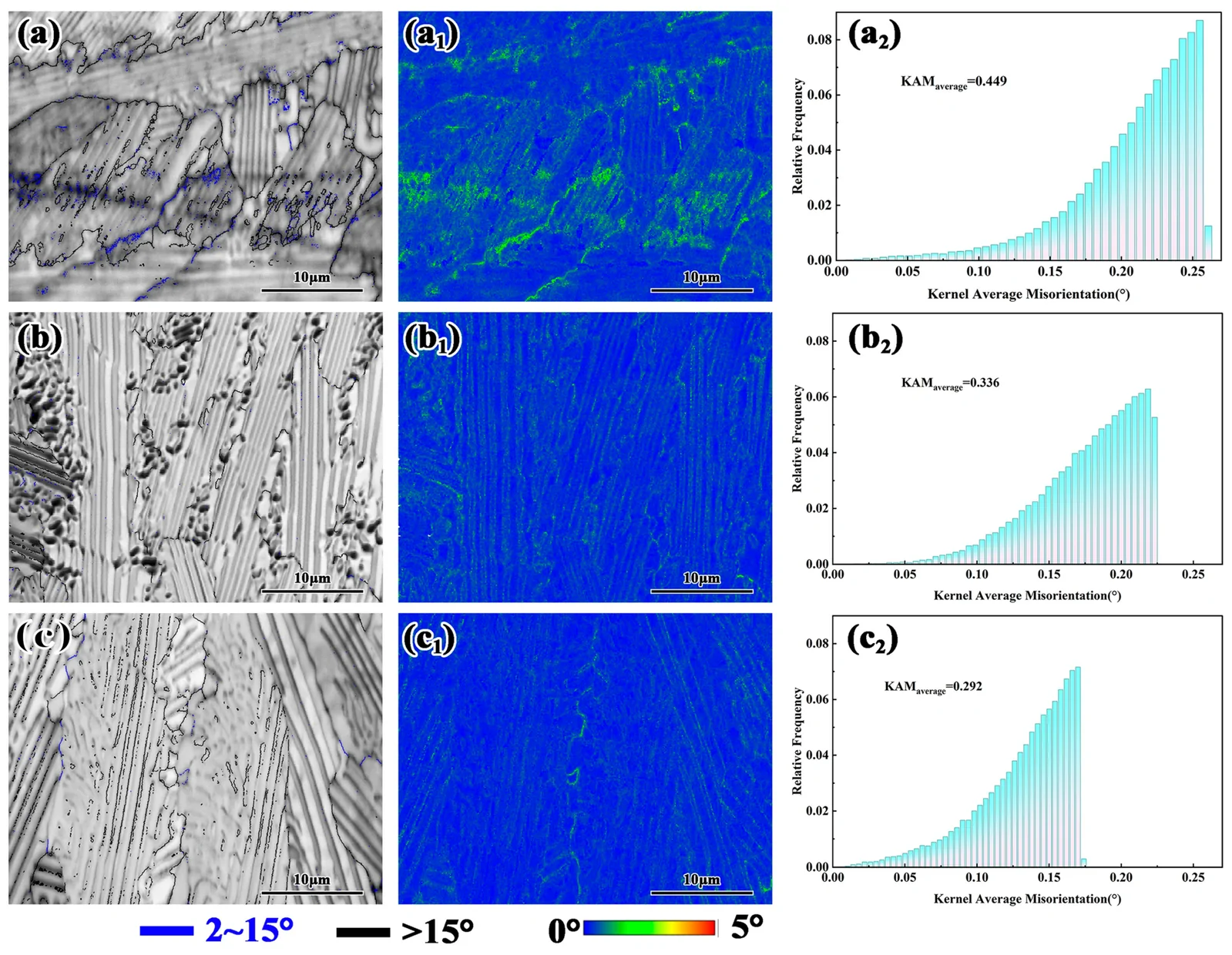
Inverse pole figure orientation maps and corresponding KAM maps of the regions containing secondary cracks in the tensile-deformed AlCoCrFeNi_2.1_ specimens fabricated at different laser powers: (**a**–**a_2_**) 1400 W, (**b**–**b_2_**) 1600 W, and (**c**–**c_2_**) 1800 W.

To further quantify the deformation heterogeneity, KAM analyses were performed for the specimens fabricated at 1400 W, 1600 W, and 1800 W, and the corresponding band-contrast and KAM maps are presented in Figure 15. Local lattice distortion was mainly concentrated near grain boundaries, eutectic-colony boundaries, and FCC/BCC phase interfaces. The average KAM value decreased systematically from 0.449° for the 1400 W specimen to 0.336° and 0.292° for the 1600 W and 1800 W specimens, respectively. The higher value measured for the 1400 W specimen indicates a greater capacity for deformation-induced dislocation storage under comparable characterization conditions. The difference in KAM response is closely related to the evolution of the eutectic lamellar spacing. The specimen fabricated at 1400 W possessed the finest FCC/BCC lamellar structure, with an average interlamellar spacing of approximately 399.1 nm. Refinement of the lamellar structure increases the interfacial area per unit volume and shortens the effective dislocation mean-free path. During tensile deformation, dislocations generated in the relatively soft FCC phase are impeded by the hard ordered BCC lamellae and accumulate near the semi-coherent FCC/BCC interfaces. The increased interface density therefore promotes extensive dislocation multiplication, interaction, and storage. In contrast, the coarser lamellar structures formed at 1600 W and 1800 W contain fewer phase boundaries per unit volume, providing fewer barriers and storage sites for mobile dislocations. This reduces the overall accumulation of geometrically necessary dislocations and is consistent with the lower average KAM values measured at higher laser powers.

The microhardness and tensile properties consequently exhibit a pronounced dependence on the lamellar scale. The specimen fabricated at 1400 W achieved the most favorable hardness and strength–ductility combination. Increasing the laser power to 1600 and 1800 W resulted in monotonic reductions in both strength and tensile ductility. The superior mechanical performance of the 1400 W specimen can be attributed primarily to the synergistic contributions of interfacial strengthening, dislocation strengthening, and hetero-deformation-induced back-stress strengthening. The refined lamellar structure produces a pronounced interface-strengthening effect. The closely spaced FCC/BCC interfaces act as barriers to dislocation glide and reduce the effective slip length within the FCC lamellae [17]. This contribution may be described qualitatively by a Hall–Petch type dependence on the characteristic lamellar spacing, according to the microhardness increases as the interlamellar spacing decreases [30]. The reduced FCC/BCC interfacial density increases the effective dislocation mean-free path and decreases the number of barriers and storage sites for dislocations, thereby lowering the resistance to plastic deformation. The semi-coherent interfaces can obstruct dislocation motion while still permitting controlled dislocation transmission or interfacial slip under sufficiently high applied stresses. Such an interfacial configuration is advantageous because it enhances strength without completely suppressing plastic compatibility between the two phases. Indeed, the high density of stored dislocations contributes to forest-dislocation strengthening. During plastic deformation, mobile dislocations multiply and interact with pre-existing dislocations, forming junctions, jogs, and tangles that impede subsequent glide. The resulting increase in dislocation–dislocation interactions continuously raises the stress required for further plastic flow. In accordance with the Taylor relationship, the dislocation-strengthening contribution increases approximately with the square root of the dislocation density [31]. The higher average KAM value of the 1400 W specimen therefore supports a stronger dislocation-hardening contribution than those of the specimens fabricated at higher laser powers.

The mechanical incompatibility between the relatively soft FCC and hard BCC lamellae can promote strain partitioning during tensile deformation. In the refined 1400 W microstructure, the high density of FCC/BCC interfaces provides abundant sites for geometrically necessary dislocation accumulation and strain accommodation. This interpretation is consistent with the higher deformation-induced KAM level of the 1400 W specimen and its ability to sustain a positive strain-hardening response to a larger true strain. The resulting interfacial strain gradients may generate hetero-deformation-induced internal stresses, and the strain partitioning can generate geometrically necessary dislocations at the FCC/BCC interfaces to maintain deformation compatibility [16,17,32,33]. The accumulated interfacial dislocations produce a long-range back stress in the FCC phase and a corresponding forward stress in the BCC phase, which suppresses further dislocation glide in the softer FCC phase, thereby increasing the macroscopic flow stress, while the forward stress promotes plastic activation in the hard BCC phase. This coupled response contributes not only to strengthening but also to sustained strain hardening by delaying premature strain localization [34,35,36,37]. The refined lamellar structure of the 1400 W specimen maximizes this hetero-deformation-induced strengthening effect because its high interface density produces a greater number of sites for geometrically necessary dislocation accumulation. Meanwhile, the ductile FCC phase can blunt and bridge cracks initiated in the relatively brittle BCC lamellae, and the frequent variation in lamellar orientation between adjacent eutectic colonies forces propagating cracks to repeatedly deflect or branch. These mechanisms increase the energy required for crack propagation and account for the simultaneous improvement in strength and ductility [38,39,40]. This interpretation is consistent with the conclusions of Sun et al. [41], who demonstrated that refined coherent or semi-coherent hetero-phase interfaces can provide effective boundary strengthening while retaining sufficient dislocation-transfer capability to achieve a favorable strength–ductility synergy. At higher laser powers, the reduced cooling rate and prolonged thermal cycle result in coarsening of the FCC/BCC lamellar structure. The consequent reduction in interface density increases the effective dislocation mean-free path, weakens interfacial dislocation storage, and strain accommodation, thereby reducing the ability to sustain work hardening. Moreover, the coarser eutectic colonies provide longer and more continuous paths for boundary-assisted crack propagation. Plastic incompatibility therefore becomes concentrated at a smaller number of grain and colony boundaries, leading to pronounced local strain accumulation, intergranular crack initiation, and premature crack coalescence. At 1800 W, possible keyhole-related defects generated by excessive heat input may further intensify local stress concentration and accelerate fracture.

### 4.2. Effect of the Lamella Structure on Corrosion Resistance

The electrochemical results reveal an overall improvement in the corrosion performance of the AlCoCrFeNi_2.1_ EHEA with decreasing laser power. Among the investigated processing conditions, the specimen fabricated at 1400 W exhibited the most positive steady-state open-circuit potential, approximately −250 mV, and the noblest corrosion potential of −244.51 mV. It also displayed the lowest corrosion current density, and the largest capacitive arc in the Nyquist plot, the highest low-frequency impedance modulus, and the broadest high-phase-angle response in the Bode plot. Although the positive shifts in OCP and corrosion potential indicate a lower thermodynamic tendency for dissolution, the enlarged impedance response provides stronger evidence of suppressed interfacial charge transfer. Taken together, these observations suggest that the surface film formed on the 1400 W specimen offered the highest electrochemical resistance and the most stable barrier response.

The superior corrosion resistance of the 1400 W specimen is more reasonably attributed to the combined effects of lamellar refinement and reduced microscale chemical heterogeneity rather than to the FCC phase fraction alone. Under otherwise identical deposition conditions, a lower laser power shortens the lifetime of the molten pool and limits thermally activated coarsening and elemental redistribution. Consequently, the fine FCC/BCC lamellae formed at 1400 W reduce the characteristic dimensions of the local electrochemical cells and interrupt the continuity of corrosion-susceptible BCC regions. In AlCoCrFeNi_2.1_ EHEA, the Al-rich and Cr-depleted BCC phase generally behaves as the local anodic constituent, whereas the comparatively Cr-rich FCC phase acts as the cathodic constituent. Preferential dissolution of the BCC phase can therefore be accelerated when coarse or interconnected regions establish persistent micro-galvanic paths [42,43,44]. Refinement of the lamellar structure, together with a more uniform elemental distribution, decreases the effective electrochemical contrast over larger distances and restricts the propagation of phase-selective corrosion. The increased density of fine interfaces may additionally facilitate the short-range transport of passivating elements and provide abundant nucleation sites for surface oxides. This can promote rapid lateral coverage of the alloy surface and reduce the occurrence of weak or discontinuous regions within the passive layer. Similar grain-refinement effects have been associated with lower passive current density, higher film resistance, and improved resistance to passive-film breakdown in CoCrNi medium-entropy alloys [45]. Thus, the broad Bode phase-angle peak and the enlarged Nyquist arc of the 1400 W specimen are consistent with a more homogeneous interfacial film and a slower charge-transfer process. Similarly, nanostructural refinement in a eutectic FeCrNiCoNb_0.5_ alloy significantly improved passivation and repassivation behavior by suppressing localized corrosion [7].

The relatively high average of the KAM value measured in the 1400 W specimen indicates enhanced local lattice curvature and is consistent with a higher relative density of geometrically necessary dislocations. Such defects may provide high-energy sites for the initial nucleation of surface oxides and accelerate the redistribution of passivating species. Previous investigations have shown that appropriately controlled compressive residual stress can decrease passive-film current and donor density, thereby restricting point-defect transport and improving resistance to localized breakdown [42,46]. The KAM measurements suggest that the 1400 W specimen possesses the highest or an optimized compressive stress (as shown in Figure 14); this effect could act synergistically with lamellar refinement and compositional homogenization. Accordingly, the best corrosion performance at 1400 W can be ascribed primarily to the suppression of persistent FCC-BCC micro-galvanic coupling and the formation of a more uniform, resistive passive interface, with higher KAM value representing a possible additional contribution.

## 5. Conclusions

In this study, AlCoCrFeNi_2.1_ eutectic high-entropy alloy was fabricated via laser-based direct energy deposition under different laser powers. The microstructural evolution, mechanical properties, and electrochemical corrosion behaviors of the as-deposited alloys were systematically investigated. The main conclusions drawn are as follows:

(1) Laser power has a significant regulatory effect on the FCC/BCC lamellar eutectic structure of the AlCoCrFeNi_2.1_ EHEA. Increasing laser power reduces the solidification cooling rate, which in turn causes simultaneous coarsening of both the FCC and BCC lamellae. The specimen prepared at 1400 W achieves the finest lamellar morphology, with an average interlamellar spacing of 399.1 nm.

(2) The mechanical properties of the alloy are strongly correlated with the lamellar feature size. As laser power increases from 1400 W to 1600 W and 1800 W, the average microhardness decreases sequentially from 337 HV to 316 HV and 309 HV. The 1400 W specimen exhibits the optimal strength–ductility synergy, with a yield strength of 737 MPa, an ultimate tensile strength of 1185 MPa, and an elongation of 15.0%. This excellent comprehensive mechanical performance benefits from the refined lamellar structure, as well as the higher stress introduced during the rapid DED-LB solidification process.

(3) The electrochemical corrosion resistance of the alloy improves with decreasing laser power. The 1400 W specimen shows the best corrosion resistance, characterized by the lowest corrosion current density of 3.60 × 10^−8^ A·cm^−2^ and the most positive corrosion potential of −244.51 mV.

## Figures and Tables

**Table 1:** Chemical composition of the AlCoCrFeNi_2.1_ High entropy alloy powder (wt.%).

Element	Al	Co	Cr	Fe	Ni
wt.%	8.67	18.71	16.21	17.49	38.91

**Table 2:** Composition of the AlCoCrFeNi_2.1_ with different laser powers of 1400 W, 1600 W, and 1800 W, respectively (wt.%).

Specimen	Phase	Al	Co	Cr	Fe	Ni
1400 W	FCC	13.25	17.18	19.04	18.80	31.73
	BCC	32.82	12.16	5.84	9.52	39.66
1600 W	FCC	8.50	19.52	19.50	21.82	30.65
	BCC	18.68	15.93	7.14	14.00	44.26
1800 W	FCC	8.34	19.23	20.36	19.88	32.19
	BCC	22.76	14.03	6.34	10.86	46.01

**Table 3:** Electrochemical parameters of EHEA specimens in 3.5 wt% NaCl solution.

Specimens	Ecorr (mV vs. SCE)	Icorr (A/cm^2^)	OCP (mV vs. SCE)
1400 W	−244.51	3.60 × 10^−8^	−253
1600 W	−280.17	6.00 × 10^−8^	−265
1800 W	−262.05	6.60 × 10^−8^	−267

**Table 4:** Equivalent circuit parameters for EIS of the alloys in 3.5 wt% NaCl solution.

Specimen	R_s_ (Ω·cm^2^)	Q_1_ (μF⋅cm^−2^)	n_1_	R_t_ (Ω·cm^2^)	R_ct_ (Ω·cm^2^)	Q_2_ (μF⋅cm^−2^)	n_2_
1400 W	8.89	6.42 × 10^−6^	0.98	8.86	1.06 × 10^6^	6.79 × 10^−6^	0.90
1600 W	7.85	7.04 × 10^−6^	0.99	7.02	7.18 × 10^5^	1.03 × 10^−5^	0.88
1800 W	8.42	8.22 × 10^−6^	0.98	8.24	5.44 × 10^5^	9.65 × 10^−6^	0.88

## Data Availability

The original contributions presented in this study are included in the article. Further inquiries can be directed to the corresponding author(s).
